# Proteome profiling of low grade serous ovarian cancer

**DOI:** 10.1186/s13048-019-0535-z

**Published:** 2019-07-17

**Authors:** Haniyeh Bashi zadeh fakhar, Hakimeh Zali, Mostafa Rezaie-Tavirani, Roya Faraji Darkhaneh, Babak Babaabasi

**Affiliations:** 1 Proteomics Research Center, Shahid Beheshti University of Medical Sciences, Tehran, Iran; 2 Proteomics Research Center, School of Advanced Technologies in Medicine, Shahid Beheshti University of Medical Sciences, Tehran, Iran; 3 Department of Gynecology, Guilan University of Medical Sciences, Rasht, Iran; 4 Department of Genetics, Reproductive Biomedicine Research Center, Royan Institute, ACECR, Tehran, Iran

**Keywords:** Proteome, Profiling, Low grade serous ovarian cancer

## Abstract

**Background:**

Serous carcinoma, the subtype of ovarian cancer has the highest occurrence and mortality in women. Proteomic profiling using mass spectrometry (MS) has been used to detect biomarkers in tissue s obtained from patients with ovarian cancer.

Thus, this study aimed at analyzing the interactome (protein-protein interaction (PPI)) and (MS) data to inspect PPI networks in patients with Low grade serous ovarian cancer.

**Methods:**

For proteome profiling in Low grade serous ovarian cancer, 2DE and mass spectrometry were used. Differentially expressed proteins which had been determined in Low grade serous ovarian cancer and experimental group separately were integrated with PPI data to construct the (QQPPI) networks.

**Results:**

Six Hub-bottlenecks proteins with significant centrality values, based on centrality parameters of the network (Degree and between), were found including Transgelin (TAGLN), Keratin (KRT14), Single peptide match to actin, cytoplasmic 1(ACTB), apolipoprotein A-I (APOA1), Peroxiredoxin-2 (PRDX2), and Haptoglobin (HP).

**Discussion:**

This study showed these six proteins were introduced as hub-bottleneck protein. It can be concluded that regulation of gene expression can have a critical role in the pathology of Low-grade serous ovarian cancer.

## Introduction

Ovarian cancer is the fifth cause of death among other cancers in American women [1]. In Iran, it is the eighth common cancer [2].

Epithelial cell tumors are the most common type of ovarian cancer, which are responsible for 90% of women’s ovarian and 1/4 genital malignancies. Epithelial ovarian cancer had various types including serous (the most common, 50% of all ovarian cancers), mucinous (15–20%), endometriosis (25–10%), clear cell (10.5%), undifferentiated (5%) and Brenner (5%). Serous cancer is usually moderate and diagnosed at lower ages and stage with a better prognosis [3].

Furthermore, serous carcinoma has the highest occurrence and mortality comprising90% of all deaths due to ovarian cancer. However, its origin and rapid progression are poorly understood [4, 5].

Frequently the lack of reliable clinical tests and the latent stages of the disease worsen most cases of ovarian cancer (68%), more than 95% of serous who are diagnosed with poor survival chance and metastatic condition [6–8]. According to reports, women diagnosed with low grade ovarian cancer have a 5-year survival rate of approximately 80–90%, but this decreases significantly to 20–30% in late-stage diagnoses [7–9]. Despite of all advances in screenings and available therapies, none of the existing screening methods facilitate prompt diagnosis and confirmation of the ovarian cancer [10] while apparently early diagnosis is a critical factor in reducing the mortalities due to ovarian cancer [11].

Based on Staging defined by the FIGO (International Federation of Gynecology and Obstetrics) system, low-grade disease including (stage I and II) describes a tumor that is localized in its original site, with no spread to lymph nodes or other body areas. The low-grade disease has the chance of a cure if the malignancy can be surgically removed successfully [12].

Many researchers have considered using CA-125 as a biomarker for early diagnosis [13, 14]. However, CA-125 is most of the times falsely negative infertile women with serous ovarian cancer and in Low grade serous ovarian cancer and CA-125 is positive in benign diseases. Thus, it is not sensitive enough for usage in general screening [15, 16].

Recent technologies have made performing complicated studies easier in order to specify the subtypes of serous ovarian cancer using genomic, transcriptomic and proteomic [17]. Specifically, proteomic profiling of serous ovarian cancer has mainly revolved around the analysis of serous ovarian cancer cell lines, tissues, and proximal fluids, urines and cyst fluid by using mass spectrometry (MS) [18–20]. Recently, the so-called, mass-spectrometry-based quantitative proteomics is the common strategy in identifying the proteins and their alterations [21, 22].

This study reports on proteomics profiling study of Low grade serous ovarian cancer by using integrate interactome (protein-protein interaction (PPI)) and (MS) data to construct and analyze PPI networks for Low grade serous ovarian cancer from controls with 100% accuracy, sensitivity, and specificity possible through panel markers.

As all previous studies have considered metastasis or high stage ovarian cancer, this study has dedicated its focus on low grade ovarian serous.

## Materials

All chemicals used in this study were purchased from Sigma-Aldrich (St. Louis, MO, USA) with exceptions noted. Criterion precast polyacrylamide gels, TGS and XT MES electrophoresis running buffers, Ready Strip™ IPG strips, mineral oil, dithiothreitol (DTT), iodoacetamide (IA), Biolytic, and urea were purchased from Bio-RAD.

### Sampling

After obtaining informed consent form, 10 healthy volunteers (women without low-grade serous ovarian cancer) entered in the study for ovarian tissue surgery; a sample size of 1 × 1 cm was taken. A part of it was transferred to the pathology lab for natural tissue confirmation. The other part was immediately transferred to the liquid nitrogen reservoir to be transmitted to the proteomics lab at the Shahid Beheshti University Clinical Projective Research Center, Tehran. Tumor sampling was conducted on 10 patients during surgery. A part of the tissue was sent to a pathology lab to be examined pathologically (confirmation of cancer). The other sample was then transferred to a liquid nitrogen tank at 96 °C for less than 2 min, and a proteomic test was sent to the proteomics lab at the Shahid Beheshti University of Medical Sciences, Tehran.

### Experimental group

At first, all specimens were examined for the level and type of cancer by an independent pathologist and then ten women with Low grade serous ovarian cancer (and 10 without) were selected for this study. We didn’t used biochemical criteria.

They were referred to the hospitals of Guilan University of Medical Sciences in Rasht from 2014 to 2015 were sampled. Examples of scientific information relevant to the study variables, without restriction of any kind, patient and personal information were used only by a specific code and were normally archived.

### Preparation

For protein extraction frozen healthy and cancerous tissues of patients under liquid nitrogen, the condition was powdered completely. The resulting powder with lubricating buffer containing Tris-HCl, magnesium chloride, EDTA and phenyl methyl sulfonyl fluoride (PMSF) and 5 mm beta-mercaptoethanol, 0.5% CHAPS, and 10% glycerol was kept in ice for 30 min. Then, the solution was centrifuged in 16,000 Ground at 4 °C for30 minutes, and protein assay was performed by Bradford technique [23]. The sample was also taken during the dewatering. After quantification of proteins, the supernatants were kept at − 20 °C until used for electrophoresis.

### Two-dimensional gel electrophoresis

In each group, 400 μg of the extracted protein was separately mixed with rehydration buffer and The pH is applied to 3 to 10 cm (IPG) strip and was passively rehydrated with above sample solution overnight at room temperature. Isoelectrofocusing (IEF) was performed by increasing the voltage From 500 to 8000 V during the first 3 h, and then a gradient pattern was used to achieve 8000 V for 3 h. Following the IEF, IPG strips were incubated equilibration buffer containing 6 M urea, 30% glycerol, 2% SDS, 2% DTT and then alkylated for 20 min in the same buffer with 2.5% iodoacetamide instead of DTT, to separate the second dimension; the treated strips were transferred onto 12% SDS-Polyacrylamide slab gel and sealed with 1% agarose. The gels run in 2.5 W each gel for 30 min and 15 W each gel as far as the blue front of thebromophenol reaches the end of the gel. The analytical gels were stained with Coomassie blue. Gels were scanned using Bio-Rad Image Scanner and Spot detection, matching, and quantitative gel analyses were carried out with Nonlinear Progenesis software.

### Protein identification by MALDI-TOF/TOF

In-gel protein digestion was performed according to Zhou et al. with minor modifications [24]. The data search was conducted on GPS Explorer (Version 3.6, AB SCIEX). Using the search engine Mascot (Version 2.2, Matrix Science, London, UK) and the International Protein Index (IPI) database (vision 3.64, 39,871 sequences, http://www.ebi.ac.uk/IPI) identify the peptides and protein identifications. The identification of the general protein was based on two or more peptides, whose ionic scores were higher than the statistical threshold. (*p* < 0.05).

### Statistical analysis

Scanned 2DE gels were analyzed by using Non-linear Prognosis Same Spot software to compare gels together and compare the spots in one statement in gels and get the density of the same spot in each of gel. To detect significant differences between the experimental groups, analysis of variance (ANOVAs) were used. A *p*-value < 0.05 was considered to be statistically significant. Statistics were presented as means ± SE. Other multivariate analyses on protein expressions use hierarchical clustering and principal components analysis.

### Bioinformatics

Identified proteins were used to determine predicted interactions with other proteins. This functional protein association network for each entry was obtained by searching “the string” online database (http://string-db.org). The sub-networks of QQPPI were constructed and visualized by Cytoscape software [25].

The following parameters were calculated to determine biologically significant nodes. Hub and bottleneck nodes were extracted from the networks in two steps; (first) In the networks, nodes with degree greater than or equal to the sum of mean and twice the standard deviation (S.D.), i.e., mean C 2*S.D. of the degree distribution, were considered as hubs [26]. (Second) We defined bottlenecks as the proteins that were in the top 5% in terms of betweenness centrality. After all the identified proteins were matched to specific processes or functions by searching the GO in CluGO/Clupedia.

## Result

**Fig. 1:**
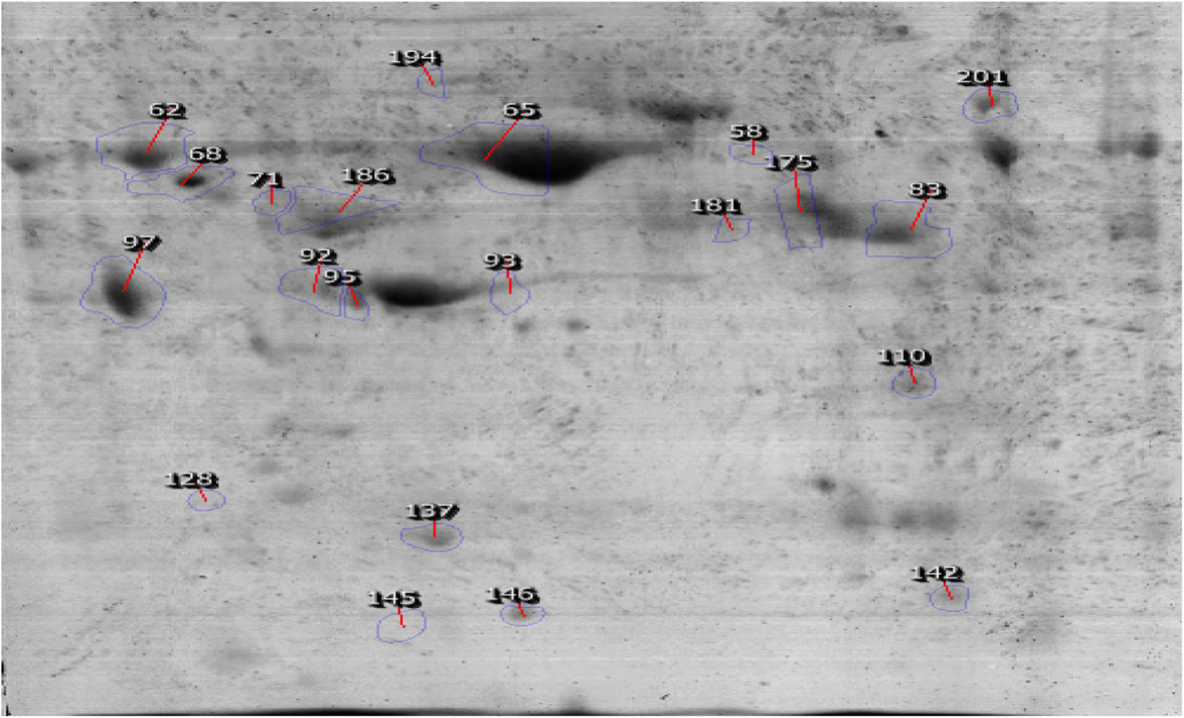
18 Protein submitted for MS-identified

**Fig. 2:**
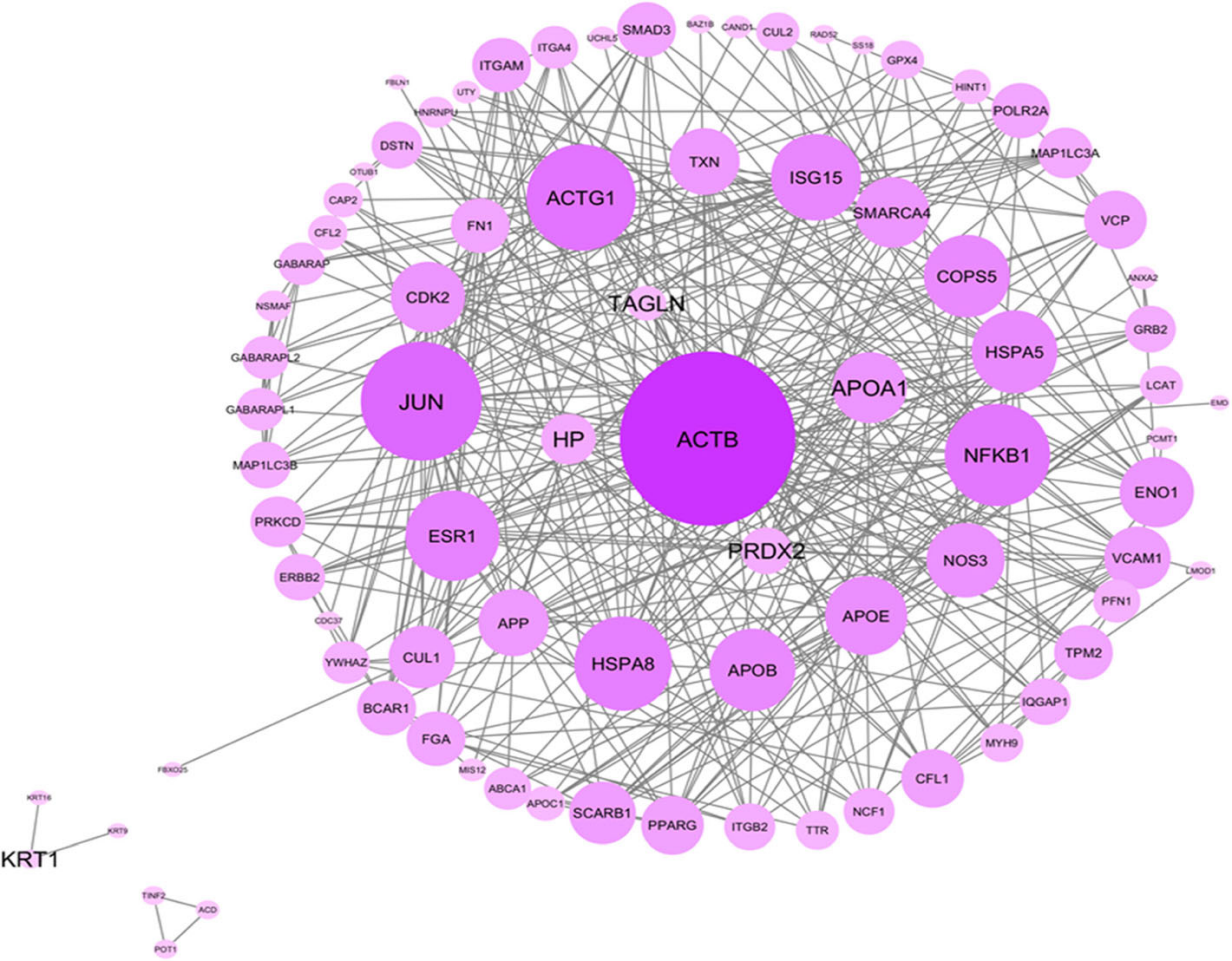
PPI network for ovarian cancer obtained from MINT, Reactome-Fls and STRING databases by the application of Proteomics Standard Initiative Common Query InterfaCe (PSICQUIC) source for the selected proteins

After ovarian tissue extraction, proteome profile of low-grade serous ovarian cancer was determined and analyzed by using Prognosis Same Spots software. The results showed that spots had a statistically significant variation with relative abundance (*p* < 0.05). As is shown in the Fig. 1, among 212 proteins 41 differentially changed expression protein (FC > 2) were identified. Among 41 spots 18 top changed expression spots were investigated by MS, and the final determined proteins are tabulated in Table 1. The 10 significant differentially expressed proteins were imported in the string, and the constructed network including 1138 nodes and 1449 edges (the network is not shown) was analyzed. The hubs, bottlenecks, and hub-bottlenecks were represented in the Tables 2, 3 and 4. For more resolution, the hub and bottleneck nodes were included in a sub-network (see Fig. 2). The finding indicates that 6 Hub-bottlenecks proteins including Transgelin (TAGLN), Keratin (KRT14), Single peptide match to actin cytoplasmic 1(ACTB), apolipoprotein A-I (APOA1), Peroxiredoxin-2 (PRDX2), and Haptoglobin (HP) (see Table 5) are query proteins which were identified by MS analysis.

**Table 1:** Proteins identified by mass spectrometry in two groups

Protein Num	Kind of Protein	Protein expression	Fold	Size	Weight Dalton
62–64,181	albumin	Decrease	2–2.1–1.1	609	69,367
201	albumin	Increase	1.8	609	69,367
83	Ig gamma-1 chain C region	Increase	1.5	334	36,500
92	Haptoglobin	Decrease	1.3	406	45,205
95	Haptoglobin	Increase	1.4	406	45,205
93	Single peptide match to actin, cytoplasmic 1	Decrease	1.7	402	41,785
110	Glyceraldehyde-3-phosphate dehydrogenase	Increase	1.8	335	36,053
137	Apolipoprotein A-I	Increase	1.6	267	30,778
142	Transgelin	Increase	1.0	201	22,611
146	Peroxiredoxin-2	Increase	1.8	198	21,892
175	Keratin	Decrease	1.5	472	51,561
186	Alpha-1-antitrypsin	Increase	1.4	503	54,030

**Table 2:** Hub proteins with significant centrality values, based on degree

Num	Hub genes	Protein name	Degree
1	ACTB	actin beta(ACTB)	913
2	APOA1	apolipoprotein A1(APOA1)	261
3	PRDX2	peroxiredoxin 2(PRDX2)	148
4	TAGLN	transgelin(TAGLN)	70
5	HP	haptoglobin(HP)	58
6	KRT1	keratin 1(KRT1)	31
7	ACTG1	actin gamma 1(ACTG1)	21
8	ERBB2	erb-b2 receptor tyrosine kinase 2(ERBB2)	16
9	CFL2	cofilin 2(CFL2)	14
10	CFL1	cofilin 1(CFL1)	13
11	LCAT	lecithin-cholesterol acyltransferase(LCAT)	12
12	APP	amyloid beta precursor protein(APP)	11
13	ESR1	estrogen receptor 1(ESR1)	11
14	APOE	apolipoprotein E(APOE)	11
15	DSTN	destrin, actin depolymerizing factor(DSTN)	11
16	NCF1	neutrophil cytosolic factor 1(NCF1)	10
17	VCAM1	vascular cell adhesion molecule 1(VCAM1)	10
18	FBXO25	F-box protein 25(FBXO25)	10
19	SMAD3	SMAD family member 3(SMAD3)	9
20	ABCA1	ATP binding cassette subfamily A member 1(ABCA1)	9
21	SMARCA4	SWI/SNF related, matrix associated, actin dependent regulator of chromatin, subfamily a, member 4(SMARCA4)	9
22	TINF2	TERF1 interacting nuclear factor 2(TINF2)	8
23	FN1	fibronectin 1(FN1)	7
24	YWHAZ	tyrosine 3-monooxygenase/tryptophan 5-monooxygenase activation protein zeta(YWHAZ)	7
25	NOS3	nitric oxide synthase 3(NOS3)	7
26	TTR	transthyretin(TTR)	6
27	JUN	Jun proto-oncogene, AP-1 transcription factor subunit(JUN)	6
28	IQGAP1	IQ motif containing GTPase activating protein 1(IQGAP1)	6
29	PFN1	profilin 1(PFN1)	6
30	HNRNPU	heterogeneous nuclear ribonucleoprotein U(HNRNPU)	6
31	POLR2A	RNA polymerase II subunit A(POLR2A)	6
32	PPARG	peroxisome proliferator activated receptor gamma(PPARG)	5
33	SCARB1	scavenger receptor class B member 1(SCARB1)	5
34	CMTM5	CKLF like MARVEL transmembrane domain containing 5(CMTM5)	5
35	HSPA8	heat shock protein family A (Hsp70) member 8(HSPA8)	5
36	NSMAF	neutral sphingomyelinase activation associated factor(NSMAF)	5
37	TRAF3IP1	TRAF3 interacting protein 1(TRAF3IP1)	5
38	SS18	SS18, nBAF chromatin remodeling complex subunit(SS18)	5
39	SYT-SSX1	Synovial sarcoma typically	5
40	CAP2	CAP, adenylate cyclase-associated protein, 2 (yeast)(CAP2)	5
41	MYH9	myosin heavy chain 9(MYH9)	5
42	EMD	emerin(EMD)	5
43	FBLN1	fibulin 1(FBLN1)	4
44	KRT9	keratin 9(KRT9)	4
45	ACD	adrenocortical dysplasia homolog(ACD)	4
46	POT1	protection of telomeres 1(POT1)	4
47	RAD52	RAD52 homolog, DNA repair protein(RAD52)	4
48	CDC37	cell division cycle 37(CDC37)	4
49	NFKB1	nuclear factor kappa B subunit 1(NFKB1)	4
50	OTUB1	OTU deubiquitinase, ubiquitin aldehyde binding 1(OTUB1)	4
51	UTY	ubiquitously transcribed tetratricopeptide repeat containing, Y-linked(UTY)	4
52	GRB2	growth factor receptor bound protein 2(GRB2)	4
53	MIS12	MIS12, kinetochore complex component(MIS12)	4
54	TXN	thioredoxin(TXN)	4
55	APOC1	apolipoprotein C1(APOC1)	4
56	FGA	fibrinogen alpha chain(FGA)	4
57	KRT16	keratin 16(KRT16)	4
58	APOB	apolipoprotein B(APOB)	4
59	NAXE	NAD(P)HX epimerase(NAXE)	4

**Table 3:** Bottlenecks proteins with significant centrality values, based on betweeness

Num	Bottleneck genes	Protein names	Betweeness
1	ACTB	actin beta(ACTB)	0.873
2	APOA1	apolipoprotein A1(APOA1)	0.222
3	PRDX2	peroxiredoxin 2(PRDX2)	0.202
4	TAGLN	transgelin(TAGLN)	0.114
5	HP	haptoglobin(HP)	0.048
6	KRT1	keratin 1(KRT1)	0.036
7	FN1	fibronectin 1(FN1)	0.026
8	APP	amyloid beta precursor protein(APP)	0.026
9	ESR1	estrogen receptor 1(ESR1)	0.026
10	UCHL5	ubiquitin C-terminal hydrolase L5(UCHL5)	0.026
11	FBLN1	fibulin 1(FBLN1)	0.017
12	TTR	transthyretin(TTR)	0.016
13	KRT9	keratin 9(KRT9)	0.016
14	VCP	valosin containing protein(VCP)	0.016
15	BAZ1B	bromodomain adjacent to zinc finger domain 1B(BAZ1B)	0.016
16	APOE	apolipoprotein E(APOE)	0.009
17	CFL1	cofilin 1(CFL1)	0.008
18	NCF1	neutrophil cytosolic factor 1(NCF1)	0.008
19	TINF2	TERF1 interacting nuclear factor 2(TINF2)	0.008
20	ACD	adrenocortical dysplasia homolog(ACD)	0.008
21	POT1	protection of telomeres 1(POT1)	0.008
22	PRKCD	protein kinase C delta(PRKCD)	0.008
23	ISG15	ISG15 ubiquitin-like modifier(ISG15)	0.008
24	ENO1	enolase 1(ENO1)	0.008
25	TPM2	tropomyosin 2 (beta)(TPM2)	0.008
26	LMOD1	leiomodin 1(LMOD1)	0.008
27	JUN	Jun proto-oncogene, AP-1 transcription factor subunit(JUN)	0.007
28	GPX4	glutathione peroxidase 4(GPX4)	0.006
29	HINT1	histidine triad nucleotide binding protein 1(HINT1)	0.006
30	VCAM1	vascular cell adhesion molecule 1(VCAM1)	0.005
31	MAP1LC3A	microtubule associated protein 1 light chain 3 alpha(MAP1LC3A)	0.005
32	MAP1LC3B	microtubule associated protein 1 light chain 3 beta(MAP1LC3B)	0.005
33	GABARAPL2	GABA type A receptor associated protein like 2(GABARAPL2)	0.005
34	GABARAPL1	GABA type A receptor associated protein like 1(GABARAPL1)	0.005
35	GABARAP	GABA type A receptor-associated protein(GABARAP)	0.005
36	PPARG	peroxisome proliferator activated receptor gamma(PPARG)	0.005
37	YWHAZ	tyrosine 3-monooxygenase/tryptophan 5-monooxygenase activation protein zeta(YWHAZ)	0.005
38	RAD52	RAD52 homolog, DNA repair protein(RAD52)	0.005
39	CDC37	cell division cycle 37(CDC37)	0.005
40	NFKB1	nuclear factor kappa B subunit 1(NFKB1)	0.005
41	OTUB1	OTU deubiquitinase, ubiquitin aldehyde binding 1(OTUB1)	0.005
42	UTY	ubiquitously transcribed tetratricopeptide repeat containing, Y-linked(UTY)	0.005
43	ITGA4	integrin subunit alpha 4(ITGA4)	0.005
44	COPS5	COP9 signalosome subunit 5(COPS5)	0.005
45	HSPA5	heat shock protein family A (Hsp70) member 5(HSPA5)	0.005
46	CUL2	cullin 2(CUL2)	0.005
47	CDK2	cyclin dependent kinase 2(CDK2)	0.005
48	CUL1	cullin 1(CUL1)	0.005
49	CAND1	cullin associated and neddylation dissociated 1(CAND1)	0.005
50	ANXA2	annexin A2(ANXA2)	0.005
51	BCAR1	BCAR1, Cas family scaffolding protein(BCAR1)	0.005
52	PCMT1	protein-L-isoaspartate (D-aspartate) O-methyltransferase(PCMT1)	0.004
53	SMAD3	SMAD family member 3(SMAD3)	0.004
54	GRB2	growth factor receptor bound protein 2(GRB2)	0.004
55	ITGAM	integrin subunit alpha M(ITGAM)	0.004
56	ITGB2	integrin subunit beta 2(ITGB2)	0.004

**Table 4:** Proteins with more hub and bottlenecks

Num	Hub and bottleneck	Protein Name	Degree	Betweeness
1	ACTB	actin beta(ACTB)	913	0.873
2	APOA1	apolipoprotein A1(APOA1)	261	0.222
3	PRDX2	peroxiredoxin 2(PRDX2)	148	0.202
4	TAGLN	transgelin(TAGLN)	70	0.114
5	HP	haptoglobin(HP)	58	0.048
6	KRT1	keratin 1(KRT1)	31	0.036
7	CFL1	cofilin 1(CFL1)	13	0.008
8	APP	amyloid beta precursor protein(APP)	11	0.026
9	ESR1	estrogen receptor 1(ESR1)	11	0.026
10	APOE	apolipoprotein E(APOE)	11	0.009
11	NCF1	neutrophil cytosolic factor 1(NCF1)	10	0.008
12	VCAM1	vascular cell adhesion molecule 1(VCAM1)	10	0.005
13	SMAD3	SMAD family member 3(SMAD3)	9	0.004
14	TINF2	TERF1 interacting nuclear factor 2(TINF2)	8	0.008
15	FN1	fibronectin 1(FN1)	7	0.026
16	YWHAZ	tyrosine 3-monooxygenase/tryptophan 5-monooxygenase activation protein zeta(YWHAZ)	7	0.005
17	TTR	transthyretin(TTR)	6	0.016
18	JUN	Jun proto-oncogene, AP-1 transcription factor subunit(JUN)	6	0.007
19	PPARG	peroxisome proliferator activated receptor gamma(PPARG)	5	0.005
20	FBLN1	fibulin 1(FBLN1)	4	0.017
21	KRT9	keratin 9(KRT9)	4	0.016
22	ACD	adrenocortical dysplasia homolog(ACD)	4	0.008
23	POT1	protection of telomeres 1(POT1)	4	0.008
24	RAD52	RAD52 homolog, DNA repair protein(RAD52)	4	0.005
25	CDC37	cell division cycle 37(CDC37)	4	0.005
26	NFKB1	nuclear factor kappa B subunit 1(NFKB1)	4	0.005
27	OTUB1	OTU deubiquitinase, ubiquitin aldehyde binding 1(OTUB1)	4	0.005
28	UTY	ubiquitously transcribed tetratricopeptide repeat containing, Y-linked(UTY)	4	0.005
29	GRB2	growth factor receptor bound protein 2(GRB2)	4	0.004

**Table 5:** Hub-bottlenecks proteins with significant centrality values, based on degree and betweeness

Protein name	Hub degree	Bottleneck Betweenees
TAGLN	70	0.144
KRT14	31	0.365
ACTB	913	0.873
APOA1	261	0.222
PRDX2	148	0.2022
HP	58	0.048

**Fig. 3:**
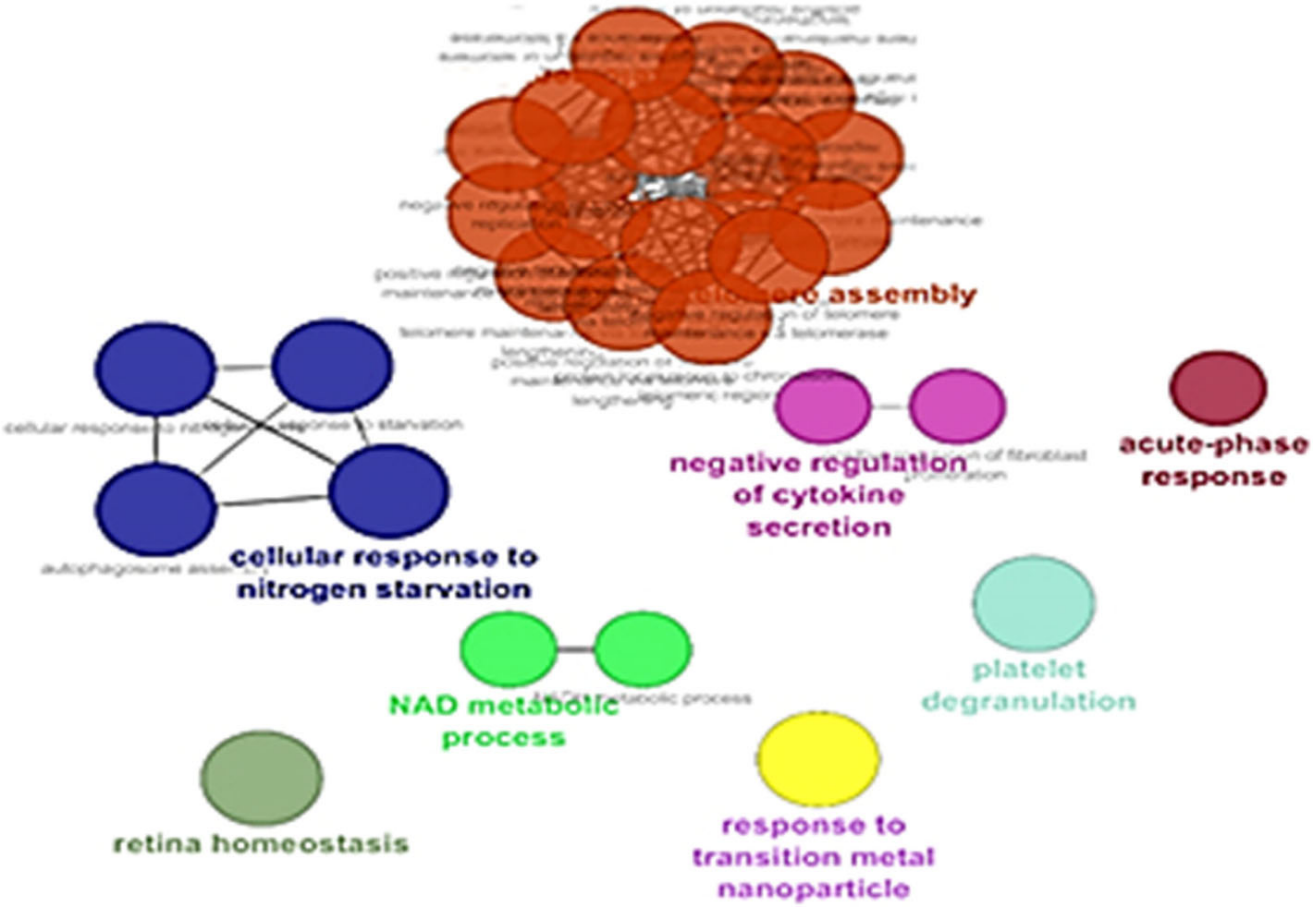
Biological process category of gene ontology analysis based on Clupedia/CluGO with identified proteins

**Fig. 4:**
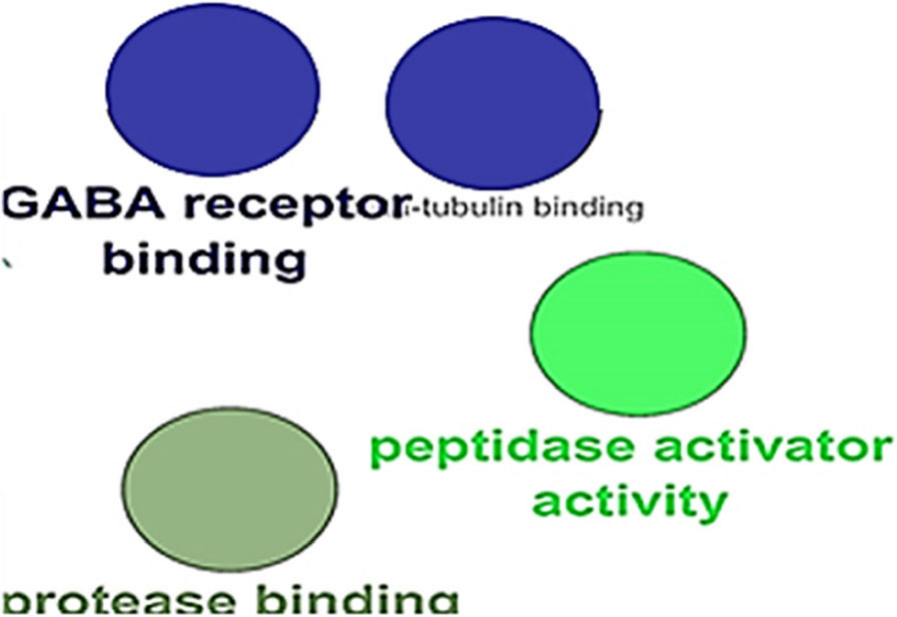
Molecular function category of gene ontology analysis based on Clupedia/CluGO with identified proteins

**Fig. 5:**
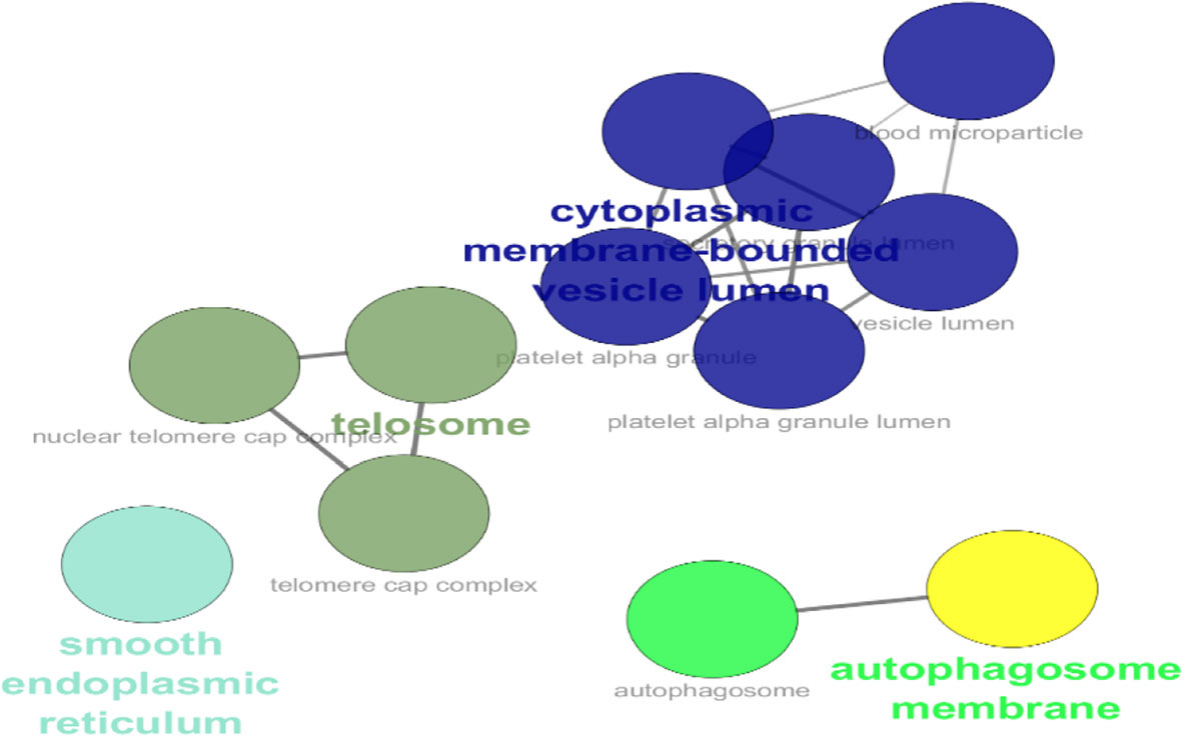
Cellular components category of gene ontology analysis based on Clupedia/CluGO with identified proteins

Gene ontology analysis counting biological processes, molecular function, and cellular component via Clupedia/CluGO were applied for the six common proteins in the central nodes and the significant differentially expressed proteins (see Figs. 3, 4 and 5).

## Discussion

Pathogenesis of serous ovarian cancer was studied by the proteomic approach to identify the alteration in gene expression between normal and low-grade cancer tissues [27]. So far, not many approaches have been devised to identify the specific differential protein expression between primary and recurrent serous in tissues of these patients [28]. For example, using quantitative proteomics via ICAT, Pan et al. compared the expression between a chemosensitive and a chemoresistant tissue [29]. Another study used paired primary and recurrent post-chemotherapy samples from high-grade serous ovarian cancer patients to identify numerous proteins elevated in recurrent tissues [30].

In our study, we found 18 proteins with different expression compared to normal and low grade of cancer tissue, which is represented in Fig. 1. There is just one study, which reported 18 metabolites differentiated in Low gradetumors in comparison to control mice, which were performed by Jones Cm et al. [11].

We constructed PPI networks of abnormally expressed proteins in the paired low garde tumor by integrating interactome and mass spectrometry data. Based on proteins which had identified by MALDI-TOF and QQPPI networks analysis, we found 6 Hub-bottlenecks proteins with significant centrality values, based on centrality parameters of the network (degree and betweenness), such as Transgelin (TAGLN), Keratin (KRT14), Single peptide match to actin, cytoplasmic 1(ACTB), apolipoprotein A-I (APOA1), Peroxiredoxin-2 (PRDX2), and Haptoglobin (HP) (Table 5).

All detected proteins in our study play an important role throughout the network because of that they all act as hubs. Transgelin is the protein according to Table 5 as the important protein in the network and overexpressed in cancer cells. Mohamed El Ayed has found Transgelin in ovarian cancer proteome [31], Zhou et al. have studied the mechanism of Transgelin in colorectal cancer, which has low endogenous levels, led to increased invasiveness, growth at low density [32]. In other human cancers such as lung adenocarcinoma, the expression of transgelin-2 has been reported. This protein has been proposed to be related to the increase in migratory and invasive abilities [33] and repressing genes involved in tumor progression [34]. In addition, several investigations have shown that Transgelin in normal and cancer cells directly interact with the actin, and alter the motility of the cells [35–38].

Keratin, has been identifies as the most commonly used marker to identify tumor cells from carcinomas and as standard detection marker for disseminated tumor cells and circulating tumor cells [39]. Cytoskeleton has a vital role in disseminated tumor cells and circulating tumor cells [40]. Keratin is one of the candidate proteins that signal the cytoskeleton [41, 42]. El Ayed reported keratin’s increasing expression in ovarian cancer [31], such as Keshamouni through analysis of human lung adenocarcinoma cell line [33]. In another study, Joosse and et al. after reviewing keratin expression during metastatic progression of breast cancer found primary breast carcinomas changes in keratin expression during metastatic progression to the lymph nodes [43].

Cytoplasmic 1 is the next protein that previously was reported by Toyama’s study on ovarian cancers [44, 45]. One study on 13 prostate cancer specimens reported cytoplasmic expression [46]. In another study on human lung adenocarcinoma cell line, this differentiation was accompanied by the modification in the expression of several cytoskeleton proteins such as cytoplasmic [33].

We identified Apolipoprotein A-I increasing expressed proteins in Low grade serous ovarian cancer such as Kristjansdottir’s study [21]. In another study about protein expression patterns associated with advanced stage ovarian cancer by Cortes, Apolipoprotein A-I was identified by their proteomic screening, which had increased expression in ovarian cancer samples [47, 48], and they suggested that protein suitable for further investigation [49]. Similarly, apolipoprotein A1 has been detected in conjunction with transthyretin and transferrin in low grade mucinous tumors [50]. ApoA-I candiminish the expression of surface molecules such as CD1a, CD80, CD86, and HLA-DR in dendritic cells, and it stimulates the production of IL -10 [51].

Furthermore, we had an increase in the expression of Ig gamma-1 chain C region in Low grade serousovarian cancer such as Cortesi study [48].

We reported peroxiredoxin-2 increasing expressions such as Kristjansdottir [21], Cortesi study [48] and Atsuhiko et al. on subtypes of ovarian carcinoma [20]. Moreover, high levels of antioxidative enzymes, such as glutathione peroxidase3, peroxiredoxin-2, peroxiredoxin-6, and superoxide dismutase, It may be responsible for resistance to apoptosis caused by oxidative stress or chemotherapy [52, 53].

One protein identified in our study was haptoglobin-1 similar to Ahmed et al. on serum of ovarian cancer patients [54]. Cortesi identified two spots by a single peptide as haptoglobin-related protein [48]. The haptoglobin is one of the richest glycoproteins secreted by the liver [55], it is reasonable to hypothesize that enhanced hepatic synthesis of the protein will occur due to an acute phase response in ovarian cancer patients resulting in elevated serum haptoglobin precursor concentration [56]. On the other hand, Haptoglobin level was shown to be affected by the amount of tumor burden and was not dependent on the histologic type or grade of ovarian malignancy [57].

On the other hand, six of the above-mentioned proteins have been identified at high grade and metastatic of serous ovarian cancer. Biton and et al. in study on high grade Bladder Tumor Transcriptome and serous ovarian cancer, characterized the luminal and basal-like subtypes of muscle-invasive bladder cancers according to the components (such as transgelin) which showed luminal tumors had lost morphological differentiation [58]. Several studies have investigated the expression of keratin in high grade ovarian cancer [59, 60]. Capo-chichi and etal in study on Overexpression and cytoplasmic localization of caspase-6 showed that it is associated with lamin A degradation in set of high grade ovarian cancers showed that it may be involved in lamin A degradation and deficiency observed in some ovarian cancer cells [61].

Many studies have reported that Apolipoprotein A-I (ApoAI) levels have been increased in early grade ovarian serous carcinoma patients [62]. Braiacu showed that several lipids (such as Apolipoprotein A) had progressive alterations in high-grade ovarian serous cancer patients with more advanced disease and poorer overall survival [63]. Elevated expression of PRDX3, PRDX5, and PRDX6 mRNAs showed poorer overall survival (OS); PRDX5 and PRDX6 also predicted poor progression-free survival (PFS) for ovarian cancer patients. Furthermore, PRDX3 played significant prognostic roles, particularly in poor differentiation and late-stage serous ovarian cancer patients [64].

In a study by Mahyuddin and etal, they demonstrated the presence of haptoglobin in ovarian cyst fluid of benign, borderline and malignant epithelial ovarian cancer. The concentration of haptoglobin was significantly raised in ES and LS-EOC compared with benign tumours. They also observed raised haptoglobin concentrations in ovarian cyst fluid of low-volume high-grade ovarian serous cancer [65].

Pathways of hub neighbors were obtained from the QUICK GO (a web-based tool that allows secure browsing of the gene ontology) [66, 67], according to STRING database information (Figs. 3, 4 and 5), related proteins of 6 bottleneck have been predicted.

On the biological process category of gene ontology analysis, we found telomere assembly as a significant factor in low-grade serous ovarian cancer. Telomeres play an important role in controlling the cell proliferation capacity [68, 69]. According to the Gray’s Study result is on ovarian cancer, Both transcriptional regulation of the human telomerase reverse transcriptase gene and alternative splicing of human telomerase reverse transcriptase transcripts can modulate the assembly of an active enzyme [70].

Cellular response to nitrogen starvation is another pathway that we found like Yoshihiro in ovarian cancer [71]. The mTOR complex 1 (mTORC1) pathway promotes cell growth in response to many cues. GATOR1 has GTPase-activating protein (GAP) activity for RagA and RagB, and its components are mutated in human cancer. In cancer cells with inactivating mutations in GATOR1, mTORC1 is hyperactive and insensitive to nitrogen starvation [72].

A key molecule that is produced due to a change in cancer metabolism reduces Nicotinamide Adenine Dinucleotide (NADH), which functions as a cofactor and provides reducing power in many enzymatic reactions that are crucial for macromolecular biosynthesis [73].NADH is also an antioxidant and forms part of the defense against reactive oxygen species (ROS) that are produced during rapid proliferation [74]. High levels of ROS can cause damage to macromolecules, which can induce senescence and apoptosis [75].

In our study, platelet degranulation plays an essential role in the biological process category of gene ontology analysis. Extensive experimental evidence shows that platelets support tumor metastasis [76]. Platelet activation and coagulation system play an important role in cancer progression [77].

According to the biological process category of gene ontology analysis, retina homeostasis may play a critical role in low-grade serous ovarian cancer. A breakthrough in Kessler’s study, understanding of the molecular biology of ovarian cancer may depend on gaining a deeper insight into retina homeostasis [78].

The biological processes are relevant by acute phase in tumor cells. The host response comprises a cascade of inflammatory signals that can be triggered by small inciting events, e.g., localized infection or a small tumor, and that leads to up- and down-regulation of a group of circulating proteins often called acute phase reactants [79]. Acute phase response in ovarian cancer earliest stages [80].

Iron is the most common metal in the human body. Epidemiological studies show that asbestos transition metal that catalyzes free radical generation is more carcinogenic [81].

Molecular function analysis showed that GABA receptor binding, peptidase activator, Protease binding are the involved function in low-grade serous ovarian cancer.

GABA receptor binding in human cancers may play a critical role in low-grade serous ovarian cancer.

MiR-224 is deregulated expression in various cancers, including cervical cancer, ovarian cancer, and lung adenocarcinoma [82].MiR-224 is located in the gamma-aminobutyric acid (GABA), a receptor epsilon gene (GABRE) [83], and its expression is directly activated by E2F1through transactivation of the GABRE gene [84].P53 and p65 bind with miR-224 host gene and inactivate the GABAA receptor ε subunit promoter in ovarian cancer [85].

Proteolytic enzymes such as peptidase have been implicated in the progression of various human malignancies, including ovarian cancer. Possibility for the role of peptidase activity in the tumor may initiate or terminate some biological events [86]. Thus, the importance of enhanced peptidase activity for malignant growth could originate from its possible regulatory role in RAS [87]. Simaga and et al. in their study on assessing the activity of peptidase activator in ovarian tissue, found that progression from benign into malignant transformation in ovarian tissue is accompanied with up-regulation of this proteolytic enzyme Such as our study [88].

Protease binding was one of the functions found inprotein-protein interaction in low-grade serous ovarian cancer in our study. Proteolytic activity is also very important at multiple stages during the intraperitoneal metastases of spheroids, especially for their initial detachment from the surface of the ovary. Since a number of published experimental studies emphasize the importance of adhesion molecules and proteases in spheroid formation, maintenance, and the subsequent adhesion of cancer cells at the secondary site, targeting their action makes biological sense [89].

Cell components of proteins are identified and based on Cytoplasmic membrane-bounded vesicle lumen, telesome, autophagosome membrane, and smooth endoplasmic reticulum.

In one study by Gilks on coexistence of intracytoplasmic lumens and membrane-bound vesicles in an invasive carcinoma, the formation of cytoplasmic lumen and membrane-bound vesicles involves two entirely separate processes that can coexist within a neoplastic cell [90].

The cell components are relevant by telesome in neoplasm cells. It is noteworthy that interference with telomeres, through direct targeting of telomeric DNA or proteins involved in the complex telomosum, can negatively affect the potential of not only tumors that express the activity of telomerase, but also those whose telomeres are transmitted through maintain unknown mechanisms [91].

In Zhen’s study on roles of the autophagosome membrane in ovarian cancer cells, They identified that autophagic cell death was reduced when cultured human ovarian cancer cells in which gene had been re-expressed were treated with growth factors, angiogenic factors, and matrix proteins found in xenografts [92] Autophagosomes then fuse with lysosomes, releasing their contents for hydrolysis [93].

The endoplasmic reticulum (ER) is responsible for the regulation of intracellular calcium (Ca2+) and the synthesis of cell surface or secretory proteins [94]. However, ER stress will induce apoptotic death if homeostatic mechanisms are insufficient to protect or repair the cell [95].

Conclusion: Biomarker discovery and molecular investigation are potent tools in the diagnosis and treatment of this disease. Protein-protein interaction network analysis increases the understanding of molecular events [96]. Here, six proteins were introduced as hub-bottleneck protein. It can be concluded that regulation of gene expression, including TAGLN, KRT14, ACTB, APOA1, PRDX2 and HP proteins can play a crucial role in the pathology of Low grade serous ovarian cancer.

## Data Availability

Not applicable.
